# Potential synergistic effect of radiotherapy and immune checkpoint inhibitors on advanced bladder cancer: A case report

**DOI:** 10.14440/bladder.2024.0075

**Published:** 2025-05-20

**Authors:** Adeel Jafri, Rachel Hubbard, Syed A. Hussain

**Affiliations:** 1 Department of Oncology and Metabolism, Sheffield Teaching Hospital, Sheffield, South Yorkshire S10 2JF, United Kingdom; 2 Department of Radiology, Sheffield Teaching Hospital, Sheffield, South Yorkshire S10 2JF, United Kingdom

**Keywords:** Bladder cancer, Immunotherapy, Radiotherapy

## Abstract

**Background:**

The combination of immune checkpoint inhibitors and radiotherapy (RT) is emerging as a promising therapeutic approach for advanced bladder cancer. However, clinical evidence of the abscopal effect in this context is still limited.

**Case presentation:**

Presented here is a 61-year-old female with high-grade urothelial carcinoma who had initially undergone chemotherapy, followed by treatment with atezolizumab (an anti-programmed death-ligand 1 antibody). Due to disease progression and symptoms, she received palliative RT alongside continued immunotherapy. Post-RT staging scans showed a significant reduction in the size of the bladder mass and a marked improvement in the patient’s quality of life. Although this case did not demonstrate a definite abscopal effect, it underscores the potential benefits of combining immunotherapy and RT.

**Conclusion:**

The observed outcomes suggest that this combination can effectively manage advanced bladder cancer, highlighting the need for further research to refine and optimize these treatment strategies.

## 1. Background

Bladder cancer treatments include surgery, intravesical therapy, chemotherapy, immunotherapy, and targeted therapy.^1^ Recent advancements emphasize multimodal approaches, such as combining radiotherapy (RT) with immune checkpoint inhibitors (ICIs), especially for advanced cases.^2^ Traditional chemotherapy offers limited survival benefits for metastatic bladder cancer, but combining RT with ICIs may improve outcomes by enhancing tumor immunogenicity and making cancer cells more susceptible to immune responses.

The abscopal effect describes how localized RT affects the treated tumor and induces tumor regression outside the radiation field. This occurs because RT can induce immunogenic cell death, releasing tumor antigens and recruiting immune cells to the tumor microenvironment. ICIs can amplify this effect by preventing tumor cells from evading immune detection. Radiation-induced tumor antigens are captured by antigen-presenting cells, such as dendritic cells, which then activate T cells to initiate a systemic immune response targeting distant tumors.^3^

Programmed death-ligand 1 (PD-L1), found on tumor and immune cells, binds to programmed cell death protein 1 on T cells, inhibiting T cell activity and allowing tumors to evade immune detection.^4^ Anti-PD-L1 antibodies block this interaction, “releasing the brakes” on T cells and enabling them to effectively target and destroy tumor cells. Combining anti-PD-L1 therapy with radiation may enhance the immune response by amplifying T cell activity.^5^ The rare occurrence of the abscopal effect in bladder cancer underscores the potential for combining RT and ICIs to trigger systemic anti-tumor responses, opening new possibilities for treating metastatic diseases.

## 2. Case presentation

A 61-year-old female presented with hematuria and right flank pain in April 2019. She was otherwise healthy, with an Eastern Cooperative Oncology Group performance status of 0, and had no risk factors for bladder cancer. A computed tomography (CT) scan revealed a tumor on the bladder wall causing right-sided ureteral obstruction, as confirmed by flexible cystoscopy. Transurethral resection of bladder tumor confirmed high-grade predominantly urothelial carcinoma with squamous differentiation invading the muscle (grade 3, stage pT2), and a CT scan of the chest confirmed mediastinal nodes.

**Figure 1:**
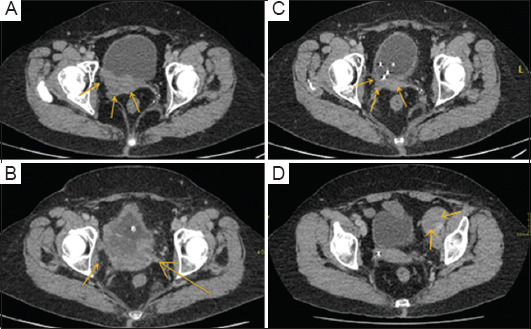
Computed tomography images taken before and after palliative radiotherapy to the bladder. (A) November 2019: Post-chemotherapy shows a large mass in the posterior bladder wall, (B) January 2020: Post-cycle two of atezolizumab shows increased size in posterior bladder wall mass, (C) May 2020: Post-radiotherapy and after 5 months of atezolizumab, the posterior bladder wall mass significantly shrank in size, (D) December 2021: End of atezolizumab scan after 2 years shows enlarged left external iliac node, estimated to be approximately 24–26 mm.

Following transurethral resection of the bladder tumor, she underwent chemotherapy (gemcitabine/cisplatin) for six cycles, which was well-tolerated. Despite this, a staging CT showed disease progression with an increase in the bladder mass size (Figure 1A). She then started a second-line palliative treatment with immunotherapy using atezolizumab in December 2019, as indicated after disease progression on chemotherapy. Atezolizumab, an anti-PD-L1 antibody, was chosen based on the National Institute for Health and Care Excellence (NICE) guidelines, which recommend its use for locally advanced or metastatic urothelial carcinoma after progression on platinum-containing chemotherapy. Atezolizumab was selected over other anti-PD-L1 antibodies due to its demonstrated clinical efficacy in improving overall and progression-free survival in locally advanced or metastatic urothelial carcinoma post-platinum-containing chemotherapy, its favorable tolerability profile, and its endorsement by NICE guidelines. Furthermore, its mechanism of action, which inhibits PD-L1 to help the immune system recognize and attack cancer cells more effectively, also played a crucial role in the decision to use it.^6^

**Figure 2:**
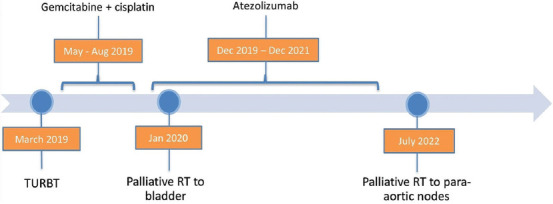
Treatment course. Transurethral resection of bladder tumor is an uncomplicated procedure, followed by palliative radiotherapy given at 20 Gy in five fractions on both occasions. Gemcitabine+carboplatin for six cycles of chemotherapy administered in a palliative setting with no interruptions to treatment. Treatment with atezolizumab (an immune checkpoint inhibitor) lasted for 2 years in a palliative setting with no disruption to treatment.

A CT scan performed in January 2020, after two cycles of atezolizumab, revealed further tumor growth in the posterior wall of the bladder (Figure 1B). The patient presented at follow-up with severe perineal pain, consistent with the local effects of the tumor growth. She was referred for palliative RT to alleviate the discomfort. The pain was later found to be caused by a large para-urethral mass representing the tumor’s inferior extremity. She received palliative RT (20 Gy in five fractions) from January 21, 2020, to January 27, 2020. Over the next 4 weeks, her pain significantly improved, and she continued with atezolizumab. Figure 2 provides a detailed timeline of the patient’s diagnosis and treatment course, emphasizing the integration of RT with ongoing atezolizumab.

A follow-up CT scan performed in May 2020 – 5 months after initiating atezolizumab and serving as the first post-RT evaluation – exhibited significant improvement in the bladder mass (Figure 1C). However, a mixed response was noted in the nodal sites: Some lymphadenopathy improved, whereas a subcarinal node and retrocaval nodes were slightly enlarged (approximately 12–15 mm). Subsequent scans every 3 months during atezolizumab treatment showed a sustained response in the bladder with no tumor recurrence.

After receiving both ICI and RT, the patient’s quality of life improved significantly, with notable shrinkage of the bladder mass. Before treatment, she suffered from considerable discomfort and struggled to sit down due to the presence of the paraurethral mass. She was rated 10 for her pain on the Visual Analog Scale. However, post-treatment, she reported significant relief from these symptoms, indicating a tangible improvement in daily functioning and comfort. Remarkably, she provided a rating of 1 on the Visual Analog Scale for her pain and has started playing golf regularly, demonstrating improved well-being. She completed 2 years of ICI treatment with atezolizumab in December 2021, showing a good quality of life and tolerability to treatment.

Ultimately, her end-of-treatment scan showed a progression of nodal disease (Figure 1D), and she decided to pursue further treatment options through clinical trials. Unfortunately, her clinical condition deteriorated in the latter half of 2022, and she passed away on October 30, 2022.

## 3. Discussion

Our case report aimed to contribute to the limited clinical evidence of abscopal effects in bladder cancer by exploring the potential synergistic interplay between ICIs and RT. The evidence presented in this case does not fully align with the accepted definition of an abscopal effect, which typically involves a systemic anti-tumor response occurring outside the radiation field. In this case, the observed regression was primarily noted in the bladder tumor, directly within the RT field, and not in distant, non-irradiated sites. Mixed responses at nodal sites, with some showing improvement in lymphadenopathy, whereas others exhibiting slight enlargement, further complicate the interpretation. These changes do not clearly reflect a systemic immunological response.

The literature on the abscopal effect in various cancers supports the notion that combining RT and ICIs can yield positive outcomes. For example, a retrospective analysis evaluating patients treated with ipilimumab for advanced melanoma revealed significantly increased median overall survival in the ipilimumab-RT arm (19 months) compared to ipilimumab alone (10 months).^7^ However, current evidence in bladder cancer remains limited, and this case alone does not provide sufficient grounds to definitively confirm the presence of an abscopal effect.

To contextualize this case further, several studies provided insights into similar multimodal approaches. A notable report described a case of advanced bladder cancer where a patient experienced significant tumor regression after receiving pembrolizumab and concurrent RT, suggesting the potential for combined treatment to enhance local control.^8^ In addition, a case series examining nivolumab and RT in metastatic urothelial carcinoma patients found improved local control and occasional systemic responses, though abscopal effects were infrequent.^9^ These findings highlight the variability in response across patients and tumor types, underlining the importance of patient selection and personalized treatment approaches.

Moreover, the BC2001 trial, a multi-center phase III study, examined the efficacy of RT with or without synchronous chemotherapy (using fluorouracil and mitomycin C) in patients with muscle-invasive bladder cancer. The results showed that combining chemotherapy with RT significantly improved locoregional disease-free survival at 2 years (67% vs. 54%) compared to RT alone, highlighting the potential benefits of combining therapies for improved local control in bladder cancer management.^10^

Collectively, these studies reinforce the growing interest in multimodal approaches for bladder cancer treatment and the need for rigorous research to optimize combinations of RT and ICIs. Despite the promising results observed in this case, more robust evidence is required to establish clear protocols and identify predictive biomarkers for response.

## 4. Conclusion

This case report highlights the potential synergy between ICIs and RT in advanced bladder cancer treatment. While the patient experienced significant tumor regression and improved quality of life after receiving palliative RT while on atezolizumab, the findings do not fully support a true abscopal effect, as a systemic tumor regression outside the irradiated field was not observed. Instead, the case underscores the need for a cautious interpretation of such outcomes and calls for further research to optimize ICI and RT combinations, focusing on factors such as timing, dose, and irradiated sites.

## Data Availability

The data will be made available upon reasonable request to the corresponding author.
